# Assessing Spatial and Temporal Patterns of Observed Ground-level Ozone in China

**DOI:** 10.1038/s41598-017-03929-w

**Published:** 2017-06-16

**Authors:** Wan-Nan Wang, Tian-Hai Cheng, Xing-Fa Gu, Hao Chen, Hong Guo, Ying Wang, Fang-Wen Bao, Shuai-Yi Shi, Bin-Ren Xu, Xin Zuo, Can Meng, Xiao-Chuan Zhang

**Affiliations:** 1 State Key Laboratory of Remote Sensing Science, Institute of Remote Sensing and Digital Earth, Chinese Academy of Sciences, Beijing, 100101 China; 2 University of Chinese Academy of Sciences, Beijing, 100101 China

## Abstract

Elevated ground-level ozone (O_3_), which is an important aspect of air quality related to public health, has been causing increasing concern. This study investigated the spatiotemporal distribution of ground-level O_3_ concentrations in China using a dataset from the Chinese national air quality monitoring network during 2013–2015. This research analyzed the diurnal, monthly and yearly variation of O_3_ concentrations in both sparsely and densely populated regions. In particular, 6 major Chinese cities were selected to allow a discussion of variations in O_3_ levels in detail, Beijing, Chengdu, Guangzhou, Lanzhou, Shanghai, and Urumchi, located on both sides of the Heihe-Tengchong line. Data showed that the nationwide 3-year MDA8 of ground-level O_3_ was 80.26 μg/m^3^. Ground-level O_3_ concentrations exhibited monthly variability peaking in summer and reaching the lowest levels in winter. The diurnal cycle reached a minimum in morning and peaked in the afternoon. Yearly average O_3_ MDA8 concentrations in Beijing, Chengdu, Lanzhou, and Shanghai in 2015 increased 12%, 25%, 34%, 22%, respectively, when compared with those in 2013. Compared with World Health Organization O_3_ guidelines, Beijing, Chengdu, Guangzhou, and Shanghai suffered O_3_ pollution in excess of the 8-hour O_3_ standard for more than 30% of the days in 2013 to 2015.

## Introduction

Ozone (O_3_) at the ground level creates a major air pollutant that affects human health^1^. High concentrations of ground-level O_3_ can cause cardiovascular and respiratory dysfunction^2–5^, and contribute to increased levels of mortality, especially for elderly people^6^. An increase of 21.3 μg/m^3^ in the mean 3-day running concentration of O_3_ resulted in a 6.6% increase in daily deaths in the warm season caused by respiratory diseases (95% CI:1.8, 11.8)^7^ in Montreal, Quebec. In China, a 10 μg/m^3^ increase of the maximum 8-h average concentration of O_3_, was reflected in increases in non-accidental mortality, cardiovascular mortality, and respiratory mortality by 0.42% (95% CI, 0.32–0.52), 0.44% (95% CI, 0.17–0.70), and 0.50% (95% CI, 0.22–0.77), respectively^8^. The World Health Organization (WHO) set a guideline of 100 μg/m^3^ for a maximum daily 8-hour average (MDA8) exposure to ground-level O_3_. Keeping air pollution below this concentration will provide adequate protection for public health, although some health effects may occur below this level^9^. The Review of Evidence on Health Aspects of Air Pollution summarized newly accumulated scientific evidence related to the adverse effects of O_3_ on human health at levels below the WHO guideline. Additionally, the Review of Evidence on Health Aspects of Air Pollution points out that O_3_ is involved in the formation of secondary inorganic and organic particulate matter (PM) in the outdoor environment. In addition, it shows that the reaction of O_3_ with common indoor volatile organic compounds (VOCs) generates a plethora of various compounds, many of which have been proposed to be respiratory irritants^10^.

Ground-level O_3_ is mainly produced during chemical reactions when mixtures of organic precursors (CH_4_ and non-methane volatile organic carbon, NMVOC), CO, and nitrogen oxides (NO_x_ = NO + NO_2_) are exposed to the UV radiation in the troposphere^11^. The most important interactions that drive the production of O_3_ concentrations in the troposphere and some of the related feedback mechanisms have been discussed thoroughly^12, 13^.

In the last few decades, the burning of biomass has been recognized as an important source of O_3_ precursors^14–16^. Because terrestrial vegetation is the dominant source of atmospheric VOCs, vegetation can have a large effect on the distribution of O_3_ and its precursors^17^. In 2008, China was recently determined to be the largest contributor to Asian emissions of CO, NO_x_, NMVOC, and CH_4_; the growth rates of these emissions from China were also the largest in Asia because of the current continuous increase in energy consumption, economic activity, and infrastructural development^18^. A complex coupling of primary emissions, chemical transformation, and dynamic transport at different scales has created the O_3_ problem^19^. In addition, the chemical transformation for O_3_ has nonlinear chemistry with respect to its precursors and the contributions from both local and regional sources^20^. The effect of VOCs and NO_x_ on O_3_ formation can be described by VOC-limited or NO_x_-limited regimes^21–23^. At elevated NO_x_ levels, which is typical of the polluted urban environment, O_3_ levels can be severely depleted locally because O_3_ reacts directly with emitted NO in a reaction known as the ‘NO_x_ titration effect.’ The rate of the process of O_3_ scavenging in the urban environment by titration with NO_x_ gradually declines as NO_x_ urban emissions are reduced when emissions are controlled. O_3_ concentrations in urban areas have increased as emissions of NO have declined. This will have an important effect on control measures and will result in an increase in the exposure of urban populations to O_3_ in the coming decade^24^.

Because of the importance of O_3_ as it relates to air quality and public health, O_3_ has received continuous attention from both the scientific and regulatory communities^25^. Numerous long-term monitoring sites have been established worldwide to observe the spatial and temporal features of ground-level O_3_. The Air Quality System of the U.S. Environmental Protection Agency contains data related to ambient air pollution collected from thousands of monitors. Regions with large urban atmospheres with poor ventilation in the Americas, such as the Los Angeles, Mexico City and Santiago de Chile metropolitan areas, have experienced O_3_ in excess of 400 μg/m^3^ for short-term ground-level O_3_ concentrations^26^. A trend analysis in Europe with the O_3_-monitoring sites data covering the 12 years from 1993 to 2005 showed that some Mediterranean cities recorded 1-hour average ground-level O_3_ concentrations exceeding 300 μg/m^3^ ^27^. The satellite remote sensing is another widely used and provides useful way to investigate the ground-level distribution of O_3_. The spatial coverage of the new generation of nadir-looking instruments onboard polar-orbiting satellites, such as the Global Ozone Monitoring Experiment, Infrared Atmospheric Sounding Interferometer and Ozone Monitoring Instrument, makes them interesting tools that can be used to monitor tropospheric O_3_ over large regions, helping researchers to assess any problems related to air quality and transport^28–31^. Nevertheless, differences still remain between a tropospheric column O_3_ derived from satellite observation and ground monitor data^32–34^.

Rapid industrialization and urbanization in China have led to high concentrations of ground-level O_3_^35^, which often cause concerns related to public health in this populous country. Although numerous studies have been conducted O_3_ epidemiology^36, 37^, such studies are less commonly available in China^38–43^. High O_3_ concentrations exceeding the national ambient air quality standards have been frequently observed in large cities of China^44–48^. Recent studies have also indicated increasing O_3_ trends exist in several highly urbanized regions of China^49, 50^. Meanwhile, few types of research have focused on the nationwide spatial and temporal variability of ground-level O_3_ concentrations in China. The Chinese government at various levels began to establish a national air quality monitoring network in 2012, which released real-time ground-level O_3_ monitoring data to the public. With the establishment of a national air quality monitoring network, large-scale real-time ground-level O_3_ monitoring data become available.

**Figure 1:**
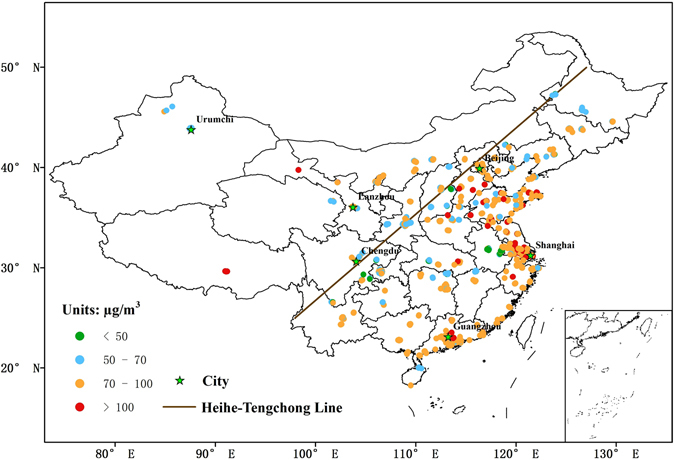
The spatial distribution of three-year averaged ground-level MDA8 ozone concentrations (μg/m^3^) during 2013–2015 and the location of 6 cities in China. We selected the same monitoring stations from 2013 to 2015, and calculated each station’s 3-year averaged ground-level ozone maximum daily 8-hour average (MDA8). This data was then imported into ArcGIS software (ArcGIS Desktop version 10.0, ESRI, Redlands, CA, USA; URL, http://www.esri.com); different concentration levels were displayed using various colors.

The spatial and temporal variability of ground-level O_3_ concentrations in China has been studied using a dataset from the national air quality monitoring network covering 2013–2015. The present paper investigates and demonstrates the spatial and temporal distribution of ground-level O_3_ on a nationwide scale, including its yearly, monthly and diurnally patterns of ground-level O_3_ concentration. In order to provide further insight into the variations between densely and sparsely populated regions, 6 major cities lied on both sides of Heihe-Tengchong line were selected to discuss in detail: Beijing, Chengdu, Guangzhou, Lanzhou, Shanghai, and Urumchi, their locations showed in Fig. 1.

## Results

### Spatial distribution of ground-level O_3_

Figure 1 shows spatial distribution of 3-year MDA8 ground-level O_3_ concentrations during 2013–2015. A total of 717 stations with fixed locations and continuous operation were selected through 2013–2015. The 3-year averaged ground-level O_3_ concentration was 80.26 μg/m^3^. Specifically, the 3-year averaged ground-level O_3_ concentrations were 81.21 μg/m^3^ and 74.35 μg/m^3^ in the densely (617 stations) and sparsely (100 stations) populated regions, respectively. Obviously, the large number of stations in the densely populated area biased the national data in favor of a larger number. The spatial distribution of averaged ground-level ozone MDA8 concentrations from 2013 to 2015 was displayed in supplemental material.

The 3-year averaged O_3_ concentrations of the densely and sparsely populated regions were analyzed as independent samples in a T-test using IBM SPSS Statistics (Table 1). The T-test for equality of means showed that the O_3_ level and variations between the densely and sparsely populated region were significantly different (Sig. <0.001 with α = 0.05).

**Table 1:** Variations in T-test results for independent samples of the three-year averaged ozone concentrations between the densely and sparsely regions.

*t*	*df*	Sig. (2-tailed)	Mean Difference	Sta. Error Difference	95% Confidence interval of the Difference	
					Lower	Upper
−4.03	715	0.00063	−6.86	1.70	−10.2	−3.51

Note: *t* is the computed T-test statistic, *df* is the degrees of freedom, Sig. (2-tailed) is the *p*-value corresponding to the given test statistic and degrees of freedom, Mean Difference is the difference between the sample mean, Std. Error Difference is the different in the standard error.

Note that a strong positive correlation between exists between surface O_3_ level and elevation^51–53^, causing the concentrations of O_3_ to often exceed recommended levels at stations located on the Qinghai-Tibet Plateau. To better visualize the situation in the densely populated region of eastern China, we used the Beijing-Tianjin-Hebei (BTH) metropolitan area along with the Yangtze River Delta (YRD) and the Pearl River Delta (PRD) regions for examples. These represent the most highly developed and populated regions in China. The 3-year mean ground-level O_3_ concentrations of BTH, YRD, and PRD were 82.14, 89.59, and 86.34 μg/m^3^, respectively.

### Temporal distribution of ground-level O_3_

**Figure 2:**
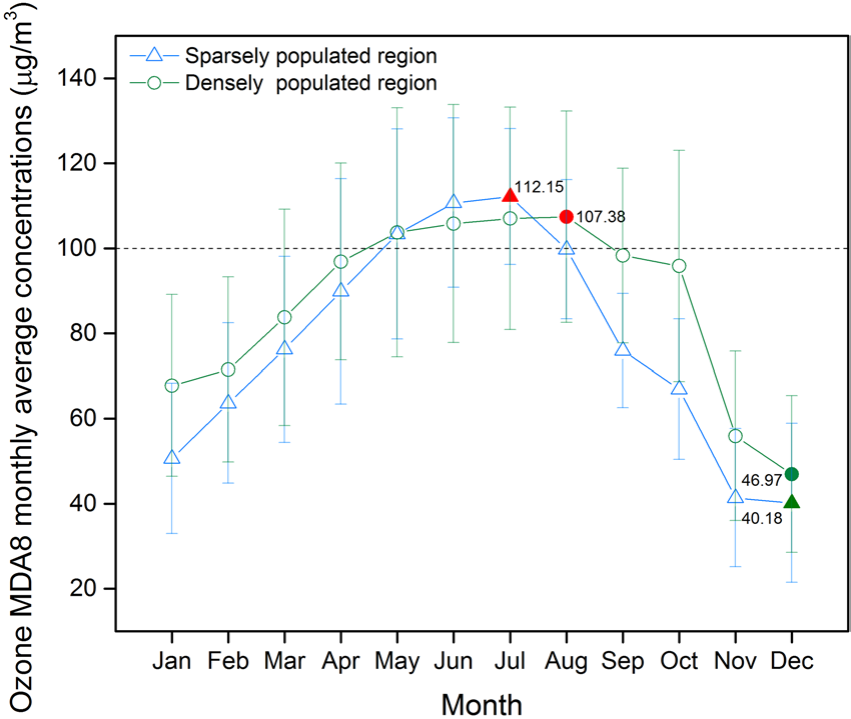
Monthly distribution of three-year averaged ground-level maximum daily 8-hour average (MDA8) ozone concentrations during 2013–2015.

Figure 2 shows mean monthly 3-year O_3_ MDA8 concentrations during 2013–2015. Generally, the monthly variability of ground-level O_3_ concentrations peaked in summer and were the lowest in winter. The peak and valley values of monthly ground-level O_3_ concentrations in the densely populated regions were 107.38 μg/m^3^ and 46.97 μg/m^3^, which were reached in August and December, respectively. In the sparsely populated region, peak and valley values were 112.15 μg/m^3^ and 40.18 μg/m^3^, and were reached in July and December, respectively.

**Figure 3:**
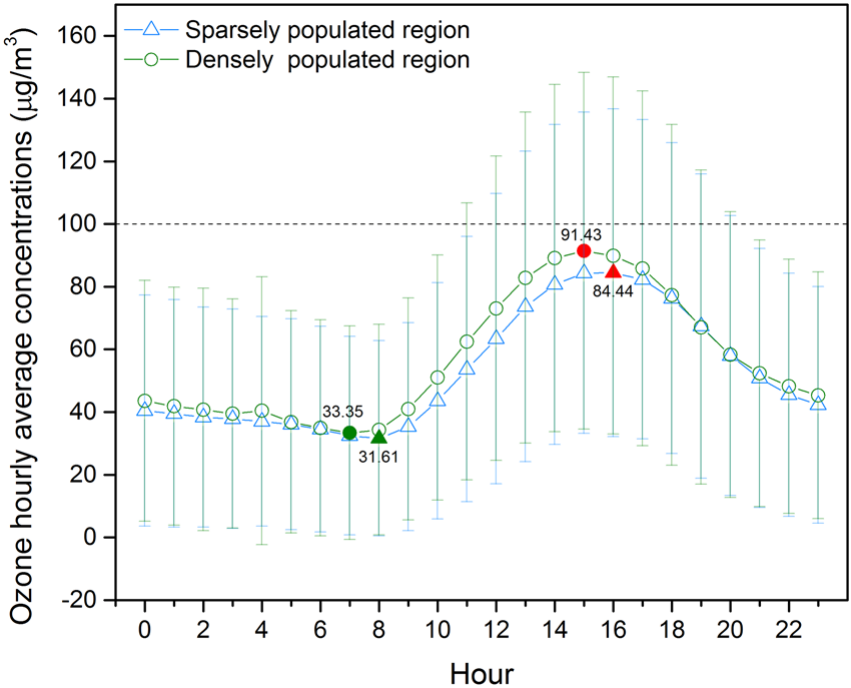
Hourly distribution of three-year averaged ground-level ozone concentrations during 2013–2015.

Figure 3 shows hourly 3-year average O_3_ concentrations during 2013–2015. Ground-level O_3_ concentrations showed a typical diurnal cycle with a minimum in the morning and a maximum in the afternoon. In the densely populated region, the maximum and minimum values of 3-year hourly averaged ground-level O_3_ concentrations were 91.43 and 33.35 μg/m^3^, and were observed at 15:00 and 7:00, respectively. In the sparsely populated region, the maximum and minimum values were 84.44 and 31.67 μg/m^3^, observed at 16:00 and 8:00, respectively.

The hourly mean concentrations of ground-level O_3_ in the densely populated region were higher than those measured in the sparsely populated region. In addition, a small peak existed at 4:00 for diurnal concentrations in the densely populated region, which was not found in the sparsely populated region; this can perhaps be explained by the accumulation of O_3_ precursors because of the relatively low boundary layer in this region^46^.

### The annual variety of ground-level O_3_ concentrations

**Figure 4:**
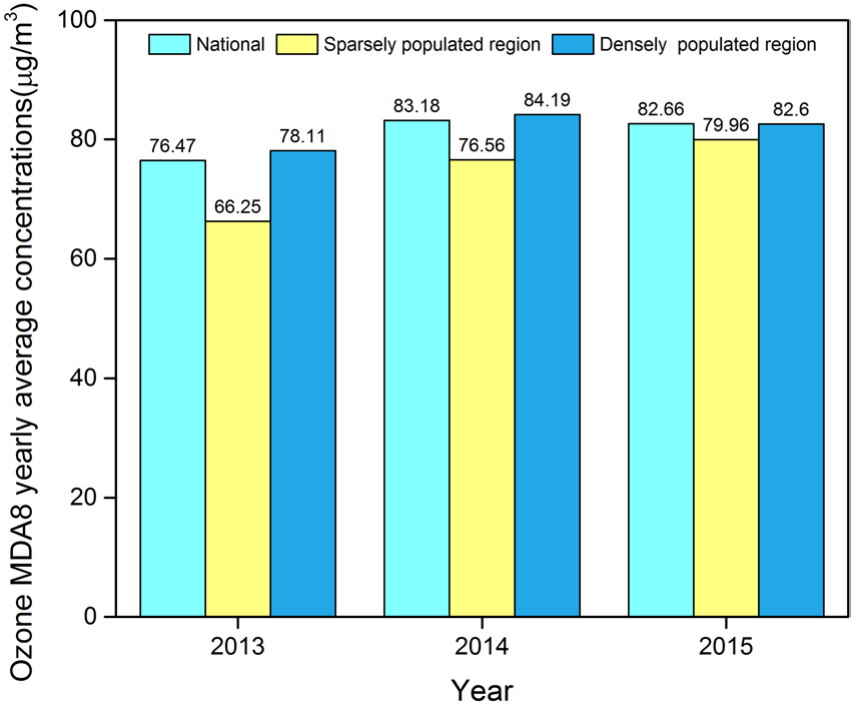
The maximum daily eight-hour average (MDA8) concentrations of annual averaged ground-level ozone in 2013–2015.

In the past 3 years, the national highest annual mean of the yearly ground-level O_3_ MDA8 concentrations from 2013 to 2015 was 83.18 μg/m^3^ in 2014, which was up by 9% when compared with 2013 (Fig. 4). For 2015, this figure was 82.66 μg/m^3^, and had remained at approximately the same level as was observed in 2014. In the densely populated region, the highest annual average concentration in the past 3 years appeared in 2014. In the sparsely populated region, the annual average concentrations of ground-level O_3_ increased year by year from 2013 to 2015.

Ground-level O_3_ is subject to *in situ* chemical reactions and physical processes that are directly affected by precursor emissions, solar radiation and other meteorological factors^54^. NO_x_ and VOCs play important roles in O_3_ formation, while NO and VOCs concentrations were not included in the data collected by the national air quality monitoring network. Therefore, yearly NO_2_ concentrations variations were measured from 2013 to 2015.

**Figure 5:**
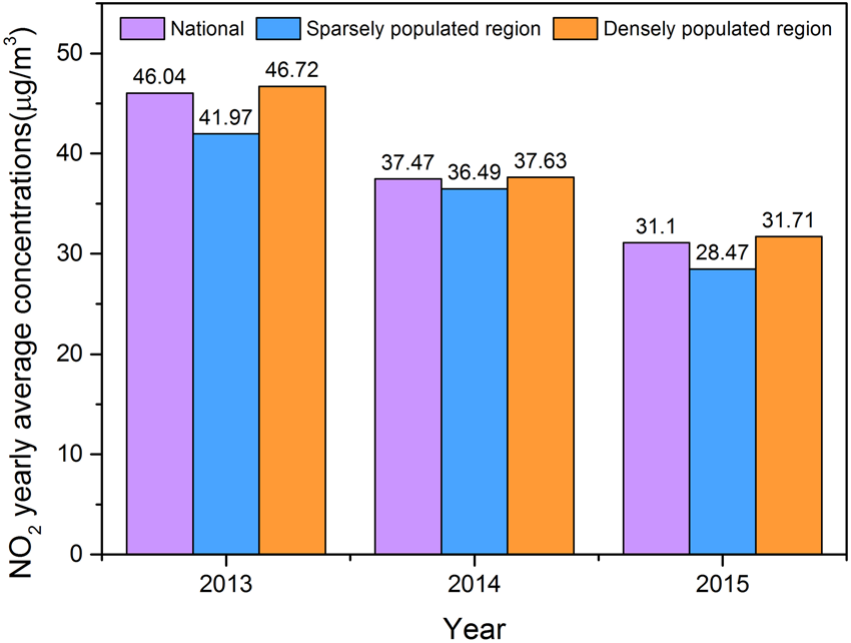
The annual averaged NO_2_ concentrations of 2013–2015.

The yearly averaged NO_2_ concentrations decreased year by year from 2013 to 2015 (Fig. 5). The national NO_2_ mean was 31.1 μg/m^3^ in 2015, and had declined by nearly 30% when compared with 2013. The Chinese State Council released the ‘Atmospheric Pollution Prevention and Control Action Plan’ on September 2013, in which they decided to implement critical strategies designed to control the burning of coal and vehicle exhaust, as well as for the management of power plants and so on^55^. After a 2-year effort, as one of the O_3_ precursors, the NO_2_ concentrations have indeed decreased since 2013^56^. In contrast, the national yearly average O_3_ concentration in 2015 was higher than that in 2013, and the increase in the ground-level O_3_ concentrations in the sparsely populated region was greater than that in the densely populated region.

The levels of O_3_ and NO_2_ are inextricably linked because of the chemical coupling of O_3_ and NO_x_. Therefore, the response to reduction in the emission of NO_x_ is remarkably non-linear^57^ and any resultant reduction in the level of NO_2_ is invariably accompanied by an increase in the level of O_3_^58^. While ground-level O_3_ formation comes from a complex coupling of primary emissions and chemical transformation, increasing the ground-level O_3_ could not only be caused by the decline in NO_2_ in China. Ma, *et al*.^54^ reported that the change of VOCs emissions might have played a more important role in the O_3_ increase than the effect of NO_x_ in the northern part of eastern China^54^. Observations have shown that Beijing’s efforts to control air pollution were somehow effective in cutting O_3_ precursors, but still left a relatively high amount of ground-level O_3_; researchers surmised that this resulted from potential contributions from VOCs and regional transport near Beijing^59^. Analysis using a smog production algorithm proved that the reduction in VOC is generally useful in reducing the photochemical production of O_3_ while the combined reduction of NO_x_ and VOC would be important to efforts to reduce the appearance of O_3_ episodes in the PRD^60^. The computation of the production rate of total oxidants (O_3_ + NO_2_) indicated that the trends of ambient oxidant levels largely depended on the ratio of VOCs/NO_x_^61^, and that a more rapid reduction in VOC reactivity would be very effective for decreasing total oxidants^59^.

**Figure 6:**
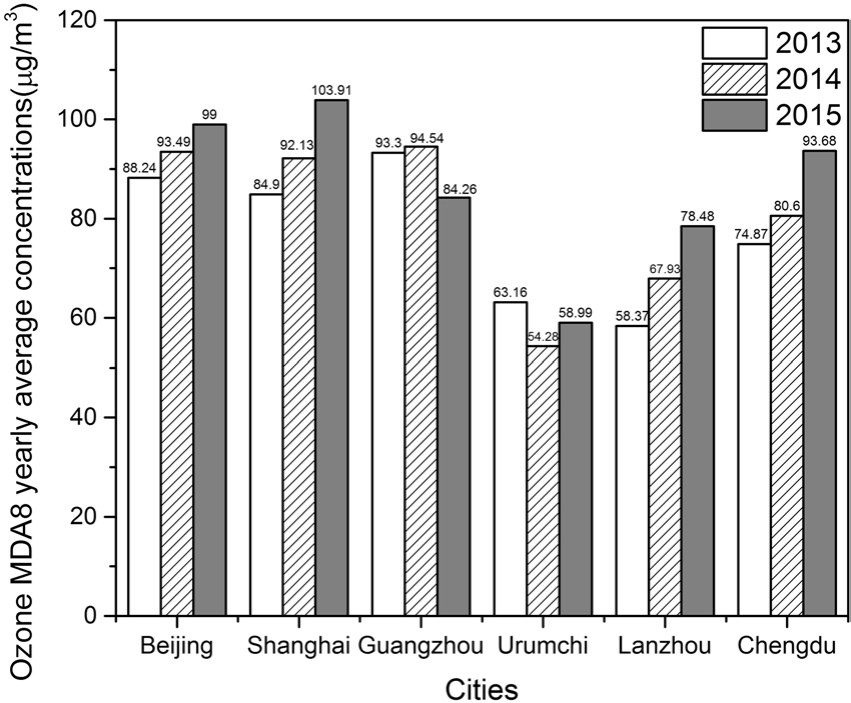
The maximum daily eight-hour average (MDA8) concentrations of annual averaged ground-level ozone in 6 cities during 2013–2015.

Figure 6 shows the yearly mean MDA8 O_3_ concentrations from 2013 to 2015 in 6 cities. Generally, yearly mean O_3_ concentrations in Beijing, Chengdu, Guangzhou, and Shanghai remained a relative high when compared with those of Lanzhou and Urumchi. Yearly average O_3_ MDA8 concentrations in Beijing, Chengdu, Lanzhou, and Shanghai in 2015 increased 12%, 25%, 34%, 22%, respectively, when compared with those in 2013. Interestingly, yearly O_3_ concentrations in 2015 decreased nearly 11% when compared with those in 2014 in Guangzhou. Except for reducing NO_x_ and CO emissions, this change also might have been caused by a pilot project launched in Guangzhou to take the lead in controlling VOC emissions that started in 2012.

**Figure 7:**
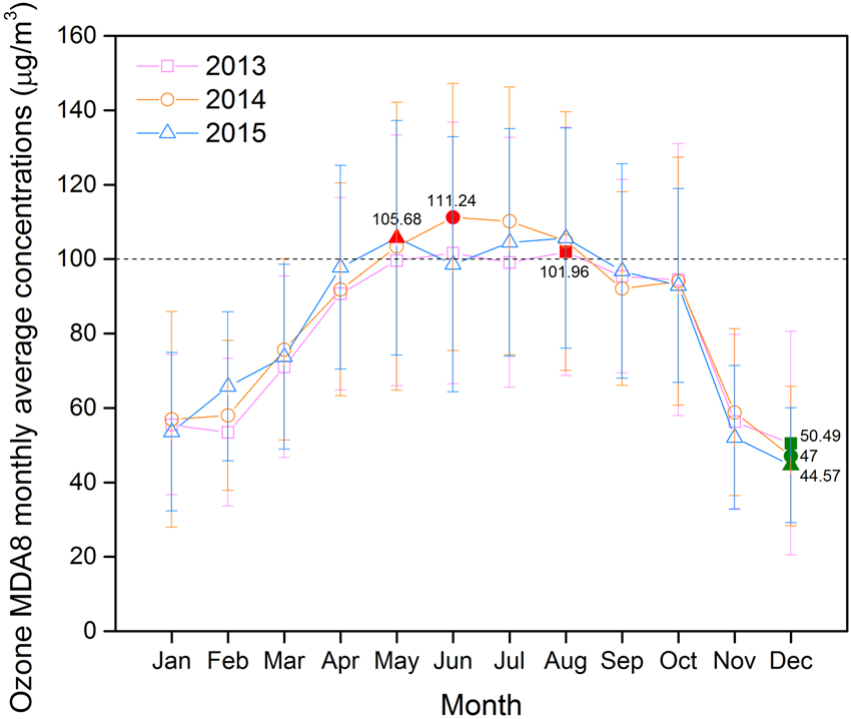
Monthly distribution of the maximum daily eight-hour average (MDA8) concentrations of ground-level ozone in densely populated regions of China during 2013–2015.

Figure 7 shows ground-level O_3_ MDA8 monthly concentrations from 2013 to 2015 in the densely populated region. The peak mean values occurred in August for 2013, June for 2014, May for 2015. The monthly mean concentration exceeded 100 μg/m^3^ during 2, 4, and 3 in 2013, 2014, and 2015, respectively, with peak monthly mean values 101.96, 111.24, and 105.68 μg/m^3^, respectively. The lowest monthly mean MDA8 values of the 3 years all occurred during December, with the values declining every year (50.49, 47.00, and 44.57 μg/m^3^ for 2013, 2014, and 2015, respectively).

**Figure 8:**
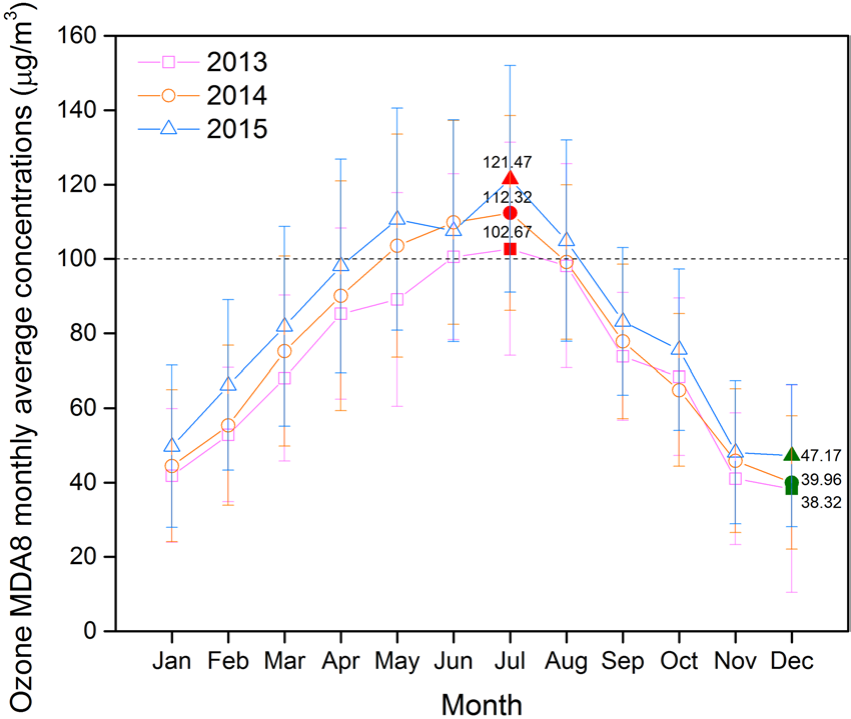
Monthly distribution of the maximum daily eight-hour average (MDA8) concentrations of ground-level ozone in sparsely populated region during 2013–2015.

Figure 8 shows the monthly ground-level MDA8 O_3_ concentrations from 2013 to 2015 in the sparsely populated region. The monthly mean peak and valley values of the 3 years in this region all occurred in July and December, respectively. In addition, the number of months with the monthly mean concentration above 100 μg/m^3^ increased from 2 to 3 to 4 months in 2013, 2014, and 2015, respectively.

**Figure 9:**
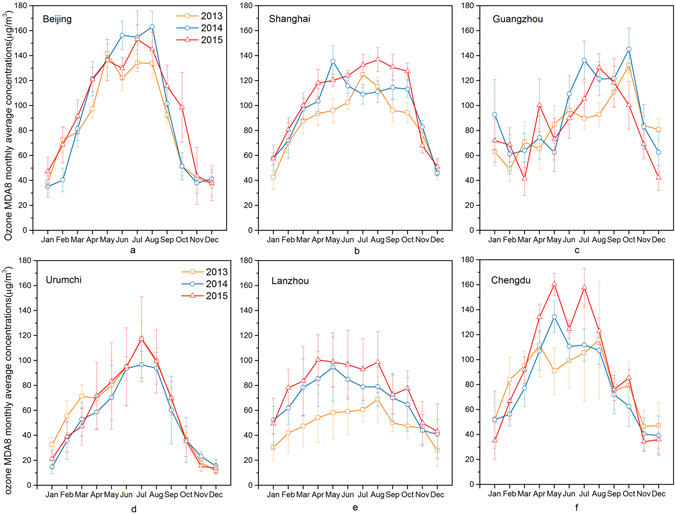
Monthly distribution of the maximum daily eight-hour average (MDA8) concentrations of ground-level ozone in six cities during 2013–2015.

Figure 9 shows the monthly mean MDA8 O_3_ concentrations from 2013 to 2015 in 6 cities. In Beijing, O_3_ concentrations remained relatively high from April to September, with lower concentrations in the winter months. O_3_ concentrations varied widely in Beijing from 2013 to 2014. The mean concentrations even peaked above 150 μg/m^3^ in August 2014 and June 2015, while the fell below 40 μg/m^3^ in December and January. In Shanghai, high O_3_ concentrations were observed from almost all of April to most of October, a little longer than occurred in Beijing. Shanghai lies in southern China and near the sea where the meteorological conditions remain suitable for O_3_ formation in October. The monthly mean concentration remained above 100 μg/m^3^ from June to August (3 months) in 2013 and from April to October in 2014 (7 months), and from March to October (8 months) in 2015. In Guangzhou, high O_3_ concentrations were observed from June to October, starting later than in Beijing and Shanghai. The highest mean O_3_ concentrations of 2013 and 2014 all occurred in October. This may have occurred because Guangzhou is located at a lower latitude and experiences a subtropical monsoon climate. Here, more sunlight and higher temperatures could favor greater O_3_ formation in summer, while typhoon rainstorms were also frequent in summer, which brought in clean ocean air that diluted the high O_3_ levels. The occurrence of fewer typhoon-related rainstorms and suitable meteorological conditions for O_3_ formation led to an increase in ground-level O_3_ concentrations in October.

In Urumchi, O_3_ concentrations remained relatively low when compared with the other 5 cities. Relatively high monthly O_3_ concentrations were observed from June to August. In November and December, the mean O_3_ concentrations can fall below 20 μg/m^3^. Urumchi is located in northwestern China at a comparatively high average elevation of 800 m. This city has stronger sunlight and less rainfall in summer than most of China, while its longer winter and lower temperatures inhibited O_3_ formation to some degree. In Lanzhou, the months of relatively high O_3_ concentrations appeared in April to August. Although O_3_ monthly mean concentrations remained below 100 μg/m^3^, the monthly and yearly mean O_3_ concentrations increased year by year from 2013 to 2015. In Chengdu, the O_3_ concentrations remained at a high level from April to August, even reaching nearly 160 μg/m^3^ in May and July 2015. Chengdu lies in the western Sichuan Basin where the western mountains often block the airflow. Affected by the basin effect, this region has poor gas diffusion capacity and high temperatures in summer. Therefore, the O_3_ accumulation easily reach a high level in summer in Chengdu.

**Figure 10:**
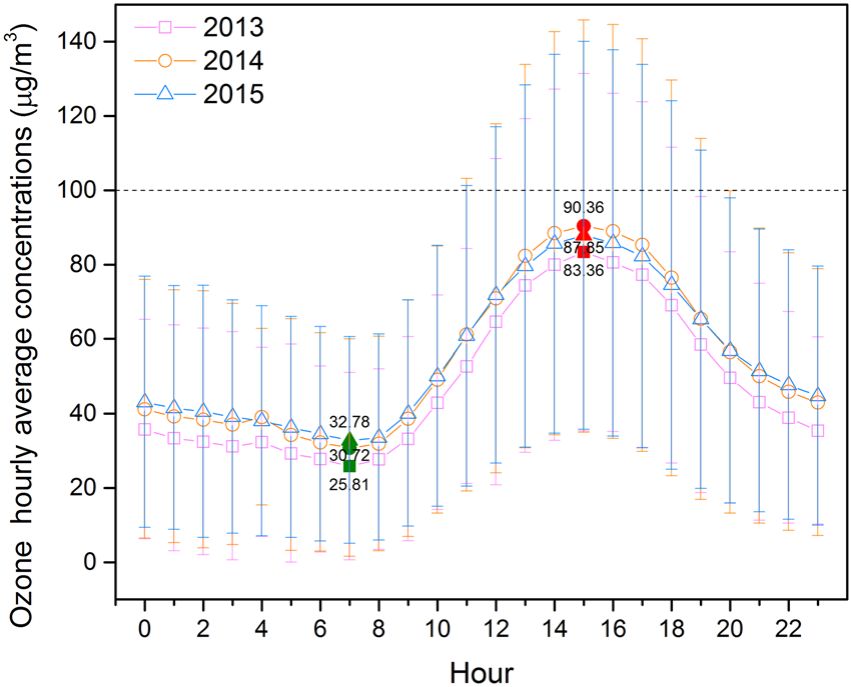
Hourly distribution of the maximum daily eight-hour average (MDA8) concentrations of ground-level ozone in densely populated regions during 2013–2015.

Figure 10 shows the hourly ground-level O_3_ concentrations from 2013 to 2015 in the densely populated region. The timing of the peak and valley values of the hourly mean concentrations of all 3 years did not change significantly in the densely populated region. These peak values were all reached at 15:00 and were 83.36, 90.36, and 87.85 μg/m^3^ for 2013, 2014, and 2015, respectively. Corresponding, the lowest values were all reached at 07:00 and were 25.81, 30.72, and 32.78 μg/m^3^ for 2013, 2014, and 2015, respectively. In the densely populated region, a smaller peak occurred at 4:00 and was not observed in the sparsely populated region.

**Figure 11:**
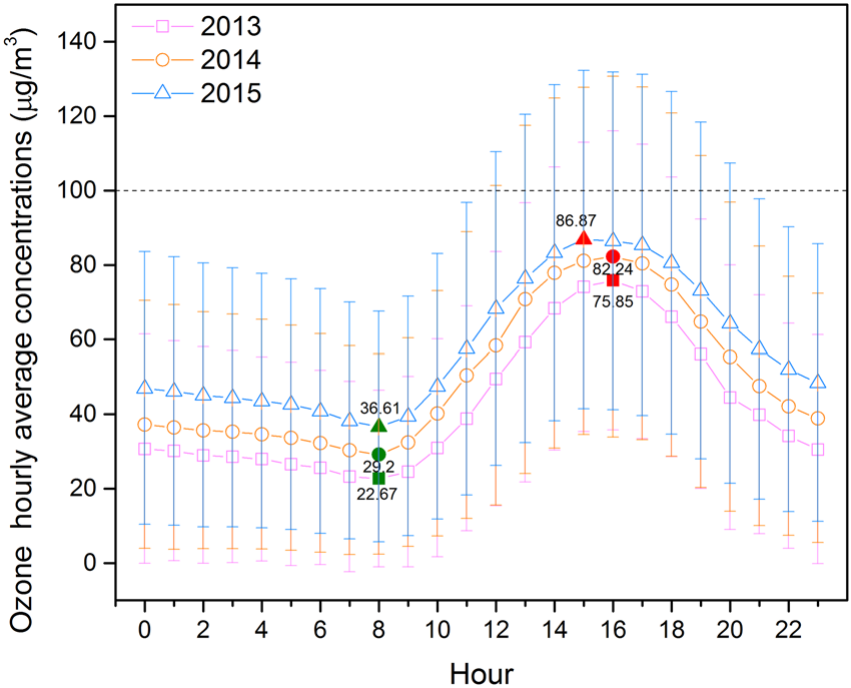
Hourly distribution of the maximum daily eight-hour average (MDA8) concentrations of ground-level ozone concentrations in sparsely populated regions during 2013–2015.

Figure 11 shows the hourly ground-level O_3_ hourly concentrations from 2013 to 2015 in the sparsely populated region. The peak values were 75.85, 82.24, and 86.87 μg/m^3^ for 2013, 2014, 2015, respectively. The valleys were 22.67, 29.2, and 36.61 μg/m^3^ for 2013, 2014, 2015, respectively. The timing of the peak values of 2015 at 15:00 were advanced by 1 hour when compared with those of 2013 and 2014 at 16:00, while timing of the valley values were consistent of 3 years at 8:00 in sparsely populated region.

**Figure 12:**
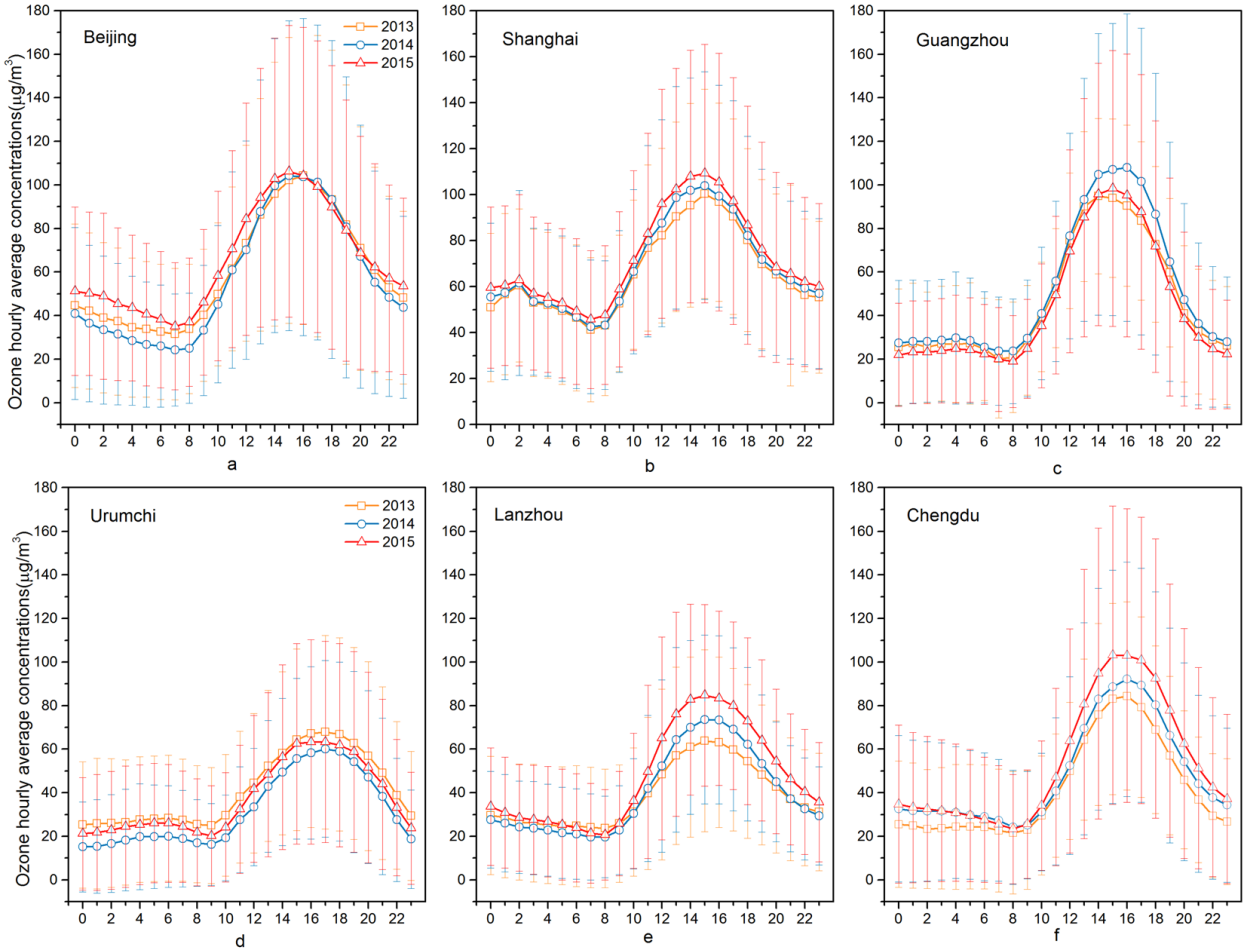
Hourly distribution of the maximum daily eight-hour average (MDA8) concentrations of ground-level ozone concentrations in six cities during 2013–2015.

Figure 12 shows the hourly mean O_3_ concentrations from 2013 to 2015 in 6 cities. As a typical product of photochemical reactions, the ground-level O_3_ concentrations was closely related to the intensity of solar radiation. The production of O_3_ began after sunrise and it accumulated until reaching peak concentrations in the afternoon. With sunset, the photochemical reactions declined rapidly to near zero without solar radiation, so that O_3_ reduction reactions occurred because of NO_x_, CO, NMHC and other O_3_ precursors, resulting in low levels of O_3_ concentrations in the night.

In Beijing, Guangzhou, and Shanghai ground-level O_3_ concentrations started to increase at almost exactly 7:00, and peaked at about 15:00. In Lanzhou and Chengdu, it began to increase at 8:00 and reached peak concentrations at around 16:00. In Urumchi, the latitude and time difference caused O_3_ concentrations to begin to increase at 9:00 and they reached peak values at 17:00. Note that all of China uses a single time zone, but spans 5 time zones of other countries, which is part of the cause of this difference.

**Figure 13:**
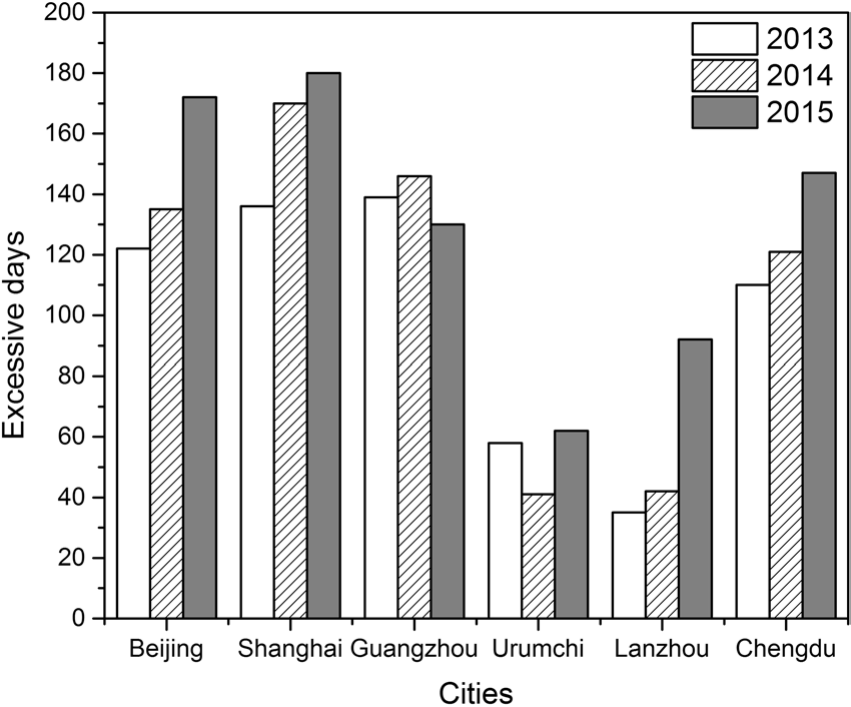
The number of days of exceeding the maximum daily eight-hour average (MDA8) concentrations of ground-level ozone in six cities during 2013–2015.

Figure 13 shows MDA8 for O_3_ exceeded the standard on some days from 2013 to 2015 in 6 cities. In Beijing, Chengdu, Lanzhou, and Shanghai, the number of days exceeding the standard when compared with the WHO O_3_ guideline (MDA8 O_3_ above 100 μg/m^3^) increased year by year from 2013 to 2015. Beijing, Chengdu, Guangzhou, and Shanghai experienced O_3_ pollution with concentrations in excess of the 8-hour standard for more than 30% of the year from 2013 to 2015. Beijing and Shanghai even suffered O_3_ pollution with concentrations in excess of the 8-hour standard for more than 45% in 2015. Corresponding to the decrease in O_3_ yearly concentration, the number of days when the standard was exceeded also declined in 2015 in Guangzhou.

## Discussion

The results from the present analysis could improve our understanding of ground-level O_3_ at a fine spatiotemporal resolution in China, given the lack of historic long-term monitoring.

The nationwide and regional three-year ground-level MDA8 O_3_ concentrations were provided above. Ground-level O_3_ concentrations showed a monthly variability peaking in summer and reaching their lowest in winter, while the diurnal cycle exhibited a minimum in the morning and peaked in the afternoon. Unlike the decrease in NO_2_ concentrations from 2013, the O_3_ concentrations began to increase after the implementation of critical emission control strategies in China. Climate, geographical location and anthropogenic emissions of precursors have caused the monthly O_3_ concentrations in different cities to vary widely. Compared with WHO O_3_ guideline, Beijing, Chengdu, Guangzhou, and Shanghai suffered O_3_ pollution in excess of the 8-hour O_3_ standard for more than 30% of the days in 2013 to 2015.

The fact that O_3_ concentrations varied on a nationwide scale and in 6 major cities after the implementation of critical air control strategies can provide decision support to future policy formulation in China. The Chinese State Council released the ‘Atmospheric Pollution Prevention and Control Action Plan’ on September 2013. Critical emission control strategies have been carried out that are designed to reduce the concentrations of particular matter smaller than 2.5 µm (PM_2.5_) and other pollutant gases. An urgent need exists for researchers to evaluate the effects of these air quality control strategies on variations in the concentration of ground-level O_3_. For 2015, report of the Chinese Ministry for Environmental Protection in 2016 showed that concentrations of PM_2.5_ decreased generally in China^57^. The present study shows that the O_3_ problem has become increasingly prominent in China. The national yearly average O_3_ concentration in 2015 was higher than that in 2013, and the increase of ground-level O_3_ concentrations in the sparsely populated region was greater than that in the densely populated region. The yearly average MDA8 O_3_ concentrations in Beijing, Chengdu, Lanzhou, and Shanghai in 2015 increased by 12% to 34%, respectively, compared with that in 2013. Beijing and Shanghai even suffered O_3_ pollution with concentrations in excess of the 8-hour standard for more than 45% of all days in 2015. With large numbers of people affected, the dramatic threat of O_3_ to public health and its wide range of influence cannot be ignored in China.

With our understanding that the complexity of the air pollution mixture in China has improved, the need for specific strategies designed to limit and control air pollution for individual pollutants has become increasingly apparent. VOCs and NO_x_ all play important roles in the formation of O_3_ at ground-level, and their effect on O_3_ is nonlinear. As a result, simply reducing levels of NO_x_ may be ineffective in managing the O_3_ problem. This, however, risks further increases in O_3_ because a VOCs/NO_x_ ratio more favorable to O_3_ production may be reached. Few long-term and nationwide observational data are available for VOCs, which only serves to limit our understanding of O_3_ production and control. It is time for the Chinese government to begin monitoring VOCs and to conduct related environmental monitoring. More specifics related to the relationships between various types of atmospheric pollution should be studied and considered during the formulation of revised management strategies.

## Methods

### O_3_ monitoring data

The Department of the Environment continuously operates and maintains the national air quality monitoring network of China, an effort that began in 2012. At each monitoring site, the concentration of O_3_ was measured using the ultraviolet absorption spectrometry method and differential optical absorption spectroscopy. The instrumental operation, maintenance, data assurance and quality control were properly conducted based on the most recent revisions of China Environmental Protection Standards^62^. The network was comprised of nearly 950 monitoring stations in 2013, which was extended to approximately 1500 stations by the end of 2015. The present study employed data from Jan. 2013 to Dec. 2015.

### Maximum daily 8-hour average O_3_

When considering the affects associated with controlled O_3_ exposures on health outcomes^63–66^, WHO set a guideline value for O_3_ exposure of 100 µg/m^3^ for a maximum period of 8 hours per day^9^. Therefore, we calculated an MDA8 for O_3_ concentration of each station. MDA8 O_3_ concentrations were calculated using greater than 5 hourly averages that were available (not zero) every 8 hours. Generally, if fewer than 6 hours of O_3_ concentration data were available for a certain 8-hour period, then this 8-hour average was assigned as the ‘missing’ value^67^. In the present study, the ‘missing’ values (zero) was not considered in the next analysis. Finally, the maximum value of the daily 8-hour averages were used as the valid MDA8.

### Delimitation of population aggregation

Heihe-Tengchong line serves as the delimitation line of population aggregation in China. The densely and sparsely populated regions were southeast and northwest of the line, respectively. About 94% of China’s population lives in 43% of the land area, creating this pattern of dense and sparse populations southeast (325.84 people/km^2^) and northwest (14.68 people/km^2^) of the line, respectively, and this pattern is not expected to fundamentally change for a relatively long time^68^.

### Average calculation

A total of 717 stations with fixed locations and continuous operation having valid MDA8 O_3_ values were selected through 2013–2015. Among the 717 stations through these 3 years, 617 and 100 stations were located in the densely and sparsely populated regions, respectively. The monthly and annual average concentrations of ground-level O_3_ were the means of the available MDA8 data from all monitoring sites in a specific area. Diurnally average concentrations were calculated using each hourly concentration of all sites. Statistical analyses were carried out using Microsoft Excel 2010 and IBM SPSS Statistics 22.

## Electronic supplementary material

Supplemental Material
