# Supplementary material for: Assessing Spatial and Temporal Patterns of Observed Ground-level Ozone in China
Source: Sci Rep. 2017 Jun 16;7:3651. doi: 10.1038/s41598-017-03929-w (PMC5473832; doi:10.1038/s41598-017-03929-w)
Supplement: Supplementary file 1 — Supplemental Material [file 41598_2017_3929_MOESM1_ESM.pdf]

Supplemental Material

Assessing Spatial and Temporal Patterns of Observed Ground-level Ozone in China

Wan-Nan Wang[1,2], Tian-Hai Cheng[1,*], Xing-Fa Gu[1], Hao Chen[1], Hong Guo[1], Ying Wang[1], Fang-Wen Bao[1,2], Shuai-Yi Shi[1,2], Bin-Ren Xu[1,2], Xin Zuo[1,2], Can Meng[1,2], and Xiao-Chuan Zhang[1,2]

[1] State Key Laboratory of Remote Sensing Science, Institute of Remote Sensing and Digital Earth, Chinese Academy of Sciences, Beijing, 100101, China

[2] University of Chinese Academy of Sciences, Beijing, 100101, China

[*]Correspondence and requests for materials should be addressed to T.H.C. (E-mail: chength@radi.ac.cn)

Figure S1: **The spatial distribution of averaged ground-level ozone MDA8 concentrations of 2013.** We calculated each station's yearly average ground-level ozone MDA8 of 2013. Then imported this data into ArcGIS software to display and distinguish different concentrations level by colors. (The map was created using ArcGIS Desktop version 10.0, the ESRI company, California, USA, URL:http://www.esri.com).

Figure S2: **The spatial distribution of averaged ground-level ozone MDA8 concentrations of 2014.** We calculated each station's yearly average ground-level ozone MDA8 of 2014. Then imported this data into ArcGIS software to display and distinguish different concentrations level

by colors. (The map was created using ArcGIS Desktop version 10.0, the ESRI company, California, USA, URL:http://www.esri.com).

Figure S3: **The spatial distribution of averaged ground-level ozone MDA8 concentrations of 2015.** We calculated each station's yearly average ground-level ozone MDA8 of 2015. Then imported this data into ArcGIS software to display and distinguish different concentrations level by colors. (The map was created using ArcGIS Desktop version 10.0, the ESRI company, California, USA, URL:http://www.esri.com).

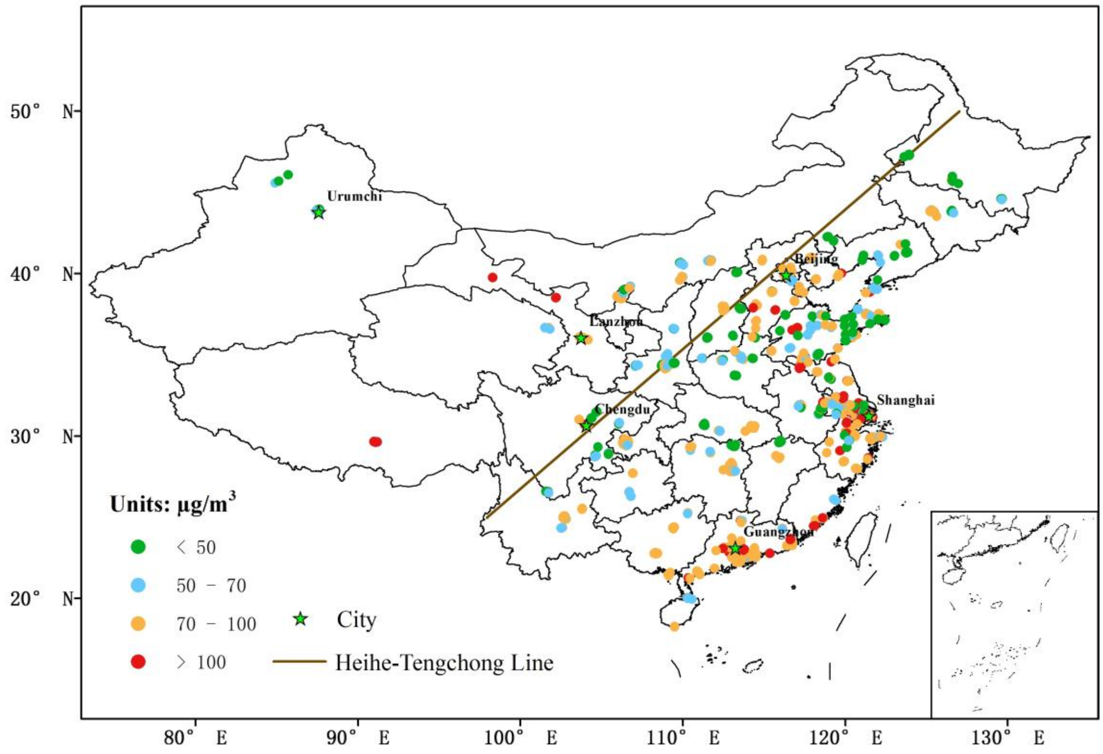

Supplementary Fig.S1

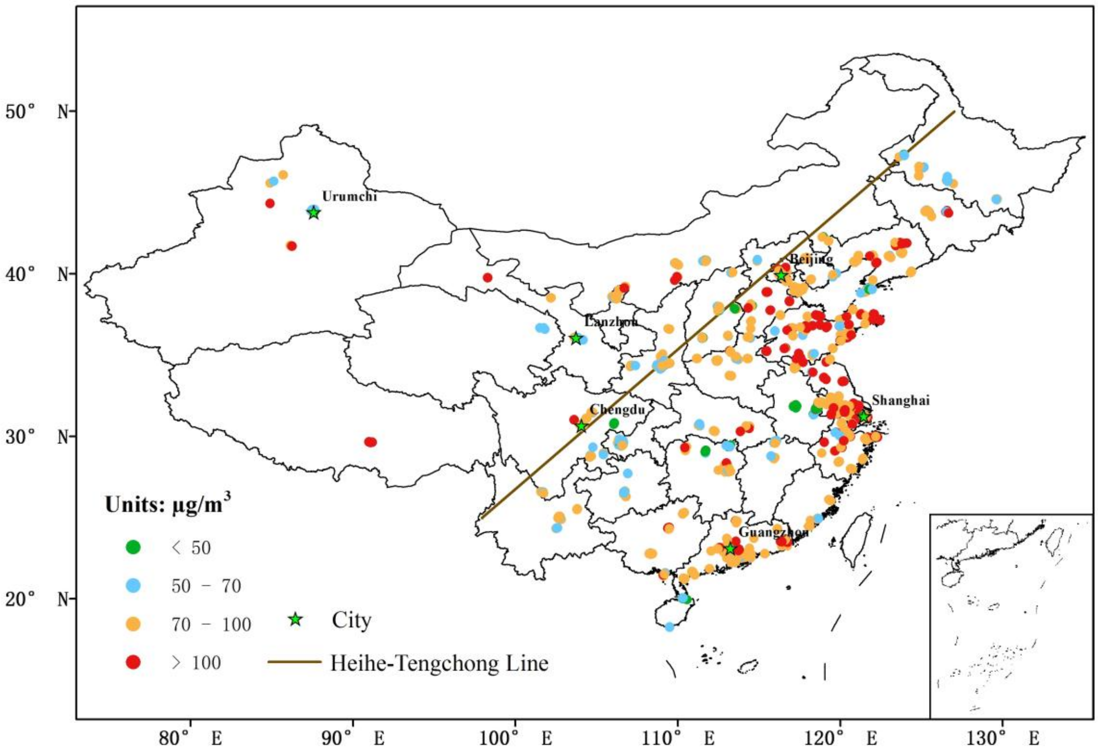

Supplementary Fig.S2

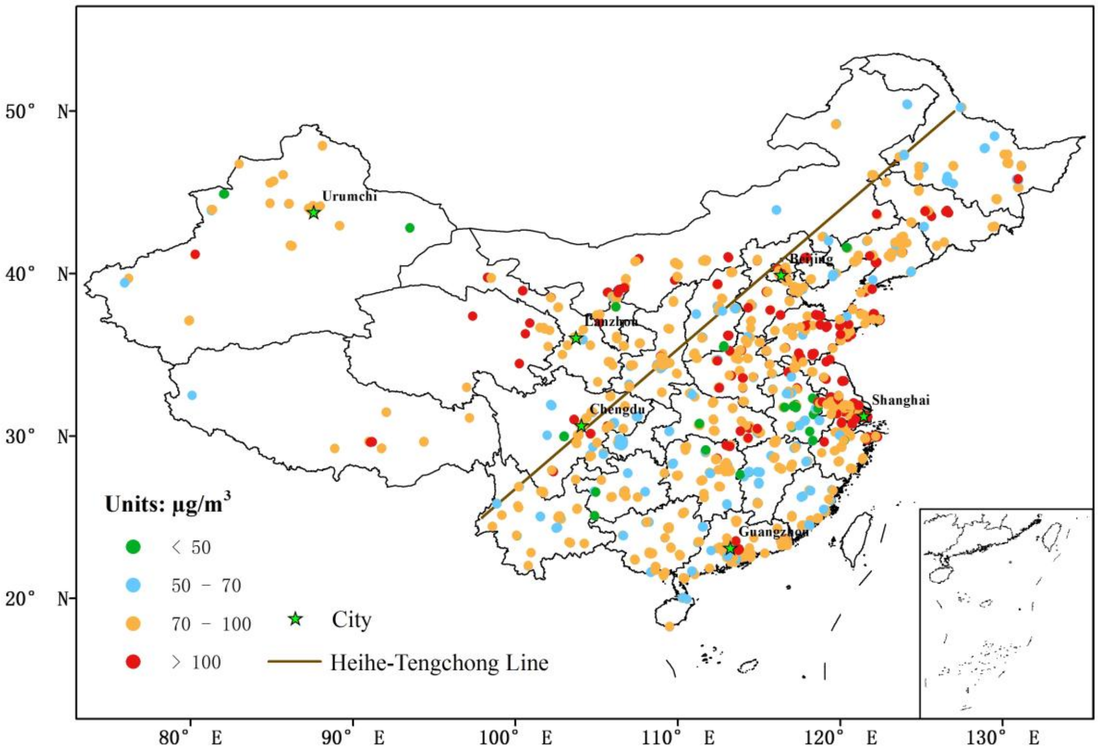

Supplementary Fig.S3
